# Reactive Oxygen Species Responsive Polymers for Drug Delivery Systems

**DOI:** 10.3389/fchem.2021.649048

**Published:** 2021-04-23

**Authors:** Fengxiang Gao, Zhengrong Xiong

**Affiliations:** ^1^ University of Science and Technology of China, Hefei, China; ^2^ Key Laboratory of Polymer Ecomaterials, Changchun Institute of Applied Chemistry CAS, Chinese Academy of Sciences, Changchun, China; ^3^ Polymer Composites Engineering Laboratory, Changchun Institute of Applied Chemistry CAS, Chinese Academy of Sciences, Changchun, China

**Keywords:** ROS, responsive polymers, nanomaterial, drug carriers, targeted delivery

## Abstract

Reactive oxygen species (ROS) play an essential role in regulating various physiological functions of living organisms; however, as the concentration of ROS increases in the area of a lesion, this may undermine cellular homeostasis, leading to a series of diseases. Using cell-product species as triggers for targeted regulation of polymer structures and activity represents a promising approach for the treatment. ROS-responsive polymer carriers allow the targeted delivery of drugs, reduce toxicity and side effects on normal cells, and control the release of drugs, which are all advantages compared with traditional small-molecule chemotherapy agents. These formulations have attracted great interest due to their potential applications in biomedicine. In this review, recent progresses on ROS responsive polymer carriers are summarized, with a focus on the chemical mechanism of ROS-responsive polymers and the design of molecular structures for targeted drug delivery and controlled drug release. Meanwhile, we discuss the challenges and future prospects of its applications.

## 

Most common cytotoxic drugs present shortcomings such as short circulation *in vivo*, poor targeting, low drug availability rates, and frequent side effects, which greatly reduce their efficacy and severely limit their clinical application. Thus, it is essential to encapsulate drug and design controlled release preparations. Fortunately, there are numerous differences in the pathological microenvironment compared to normal tissue, such as enzyme levels, pH, temperature, and ionic strength; among these factors, the most remarkable are the differences in reactive oxygen species (ROS) levels (De Palma et al., 2017). A significant increase in metabolite levels or inflammatory signals could lead to an increase in ROS levels (D’Autreaux and Toledano, 2007). For example, the concentration of H_2_O_2_ in normal tissues is strictly controlled at about 20 nmol L^−1^, while the concentration of H_2_O_2_ in tumor tissues increases up to 50–100 mol L^−1^. ROS levels in tumor cells are significantly higher than those in normal cells (Schumacker, 2015). In addition, increased ROS levels are associated with cardiovascular and cerebrovascular diseases (atherosclerosis and hypertension), diabetes, and neurodegenerative diseases (Alzheimer’s disease). Thus, ROS are ideal markers for active targeting and inducing the controlled release of drugs (Yang et al., 2019). The development of a ROS-responsive drug delivery system is very important and has excellent prospects for application in the biomedicine field (Trachootham et al., 2009; Song et al., 2014).

Nanodrugs based on currently available polymer carriers present the numerous advantages compared with inorganic substances and small molecules. Polymer carriers exhibit better biocompatibility and generally do not contain heavy metal elements. It is possible to introduce specific functional groups or multiple groups on the polymer chains, and formulate multifunctional targets to achieve personalized therapy. Moreover, different nanostructures, such as vesicles, solid nanomaterials, and porous structures, could be designed to enhance the drug-loading capacity based on the properties of drugs. Finally, and most importantly, special functional groups could be introduced to achieve targeted responses at lesion locations, such as ROS, to significantly inhibit the uncontrolled release of drugs and to increase the therapeutic effects while reducing toxicity and side effects.

Currently, the synthesis of ROS-responsive polymer carriers and their medical applications have attracted great interest from many researchers. A large number of ROS-responsive polymers for drug delivery systems have been developed and have achieved superior and encouraging therapeutic effects over traditional drugs (Lee S. H. et al., 2013; Lou et al., 2015; Saravanakumar et al., 2017; Owen et al., 2018). In this review, we discuss current progress in ROS-responsive polymers for drug delivery, including the types of ROS-responsive polymers and the advantages of application of different ROS-responsive polymers, which is determined by structural characteristics. Further, we describe the design of molecular structures of these ROS-responsive polymers. Finally, we propose future perspectives and the trends in the development of ROS-responsive polymers for drug delivery.

## Reactive Oxygen Species–Responsive Polymers for Drug Delivery

ROS is a biochemical term used to describe the chemical species formed from incomplete reactions with oxygen (D’Autreaux and Toledano, 2007; Nosaka and Nosaka, 2017). ROS mainly include hydrogen peroxide (H_2_O_2_), superoxide (O_2_•¯), hydroxyl radical (•OH), peroxynitrite (ONOO¯), singlet oxygen (^1^O_2_), and hypochlorite (OCl¯) (Gligorovski et al., 2015; Hayyan et al., 2016). ROS play a crucial role in living organisms, including the modulation of protein function, production of several hormones, regulation of cell signaling, and mediation of inflammation. However, overproduction of ROS could give rise to oxidative stress leading to serious damage of cells associated with different diseases, such as cancer (Liu H. et al., 2018), neurodegenerative diseases (Barnham et al., 2004), and inflammation (Fraisl et al., 2009).

ROS are an important feature distinguishing pathological from healthy tissues. ROS are overexpressed in diseased cells, and this property has been exploited to develop ROS-responsive drug carriers for targeted therapy (Chen X. et al., 2016; Deng et al., 2017; Dharmaraja, 2017; Lu et al., 2017). Different types of ROS-responsive polymer carriers had been explored, including thioether-containing polymers, poly(thioketal), selenium/tellurium containing polymers, arylboronic acid/ester-containing polymers, aryl oxalate esters, and poly(proline) (Cheng et al., 2011; Chen Y. et al., 2016; Xu et al., 2016; Luo et al., 2018; Sun et al., 2018; Xu et al., 2018b; Sun et al., 2019; Eleftheriadou et al., 2020; Liu et al., 2020). These ROS-responsive polymers usually break chemical bonds and/or lead to transformations from hydrophobic to hydrophilic phases, favoring the release of carrier drugs.

**FIGURE 1:**
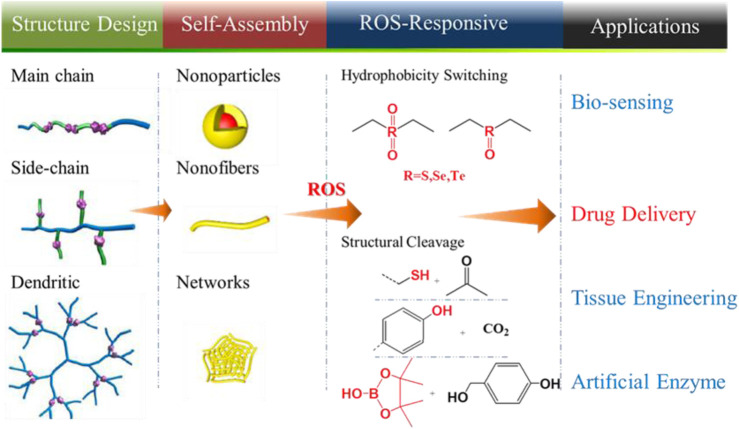
Structure design and technological process of ROS-responsive polymers for drug delivery systems (Hu et al., 2020). Copyright 2020, the American Chemical Society.

The chemical structure and the molecular weight determine the physical properties of polymers and include the glass transition temperature (*T*_*g*_), solubility, thermal stability, degree of crystallinity, and physical properties. Therefore, the design of a polymer is crucial for ROS-responsive polymers to ensure controlled release of carrier drugs. For example, the hydrophobicity of a polymer is normally increased as the molecular weight increases, while polymers with low molecular weight are always degraded more rapidly. Thus, the release rate of the drugs could be controlled by adjusting the molecular weight of the polymer. From a chemical structure standpoint, the release rate of drugs also could be regulated by increasing the water solubility of polymers through grafting or by copolymerizing hydrophilic chains onto hydrophobic polymers. Overall, when designing the chemical structure of ROS-responsive polymer carriers, the following principles should be considered: (1 good biocompatibility and easy functionalization, (2 solubility in water or in hydrogels is required to increase utilization, and (3 long half-life and does not easily accumulate in the body. The chemical structures and technological process of ROS-responsive polymers are summarized in Table 1 (Ye et al., 2019) and in Figure 1 (Hu et al., 2020), respectively. Polymers could be designed to integrate ROS-responsive chemical groups into polymers as their main chains or backbone, side chains, or tail chain, and a variety of structures of polymer (the linear copolymer, comb polymers, and dendritic copolymers) are possible. These ROS-responsive polymers could be transformed into nanoparticles, nanofibers, or hydrogels *via* self-assembly, which are widely used in biosensors, tissue engineering, and as artificial enzymes. An important and promising application is ROS-responsive polymer drug carriers.

**TABLE 1:** ROS-responsive polymer structures and mechanisms of activity (Ye et al., 2019). Copyright 2019, the American Chemical Society.

	Structure	Oxidation
Hydrophobic to hydrophilic phase transition		
Thioether Selenium Tellurium	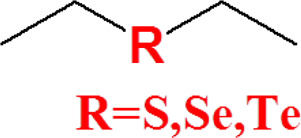	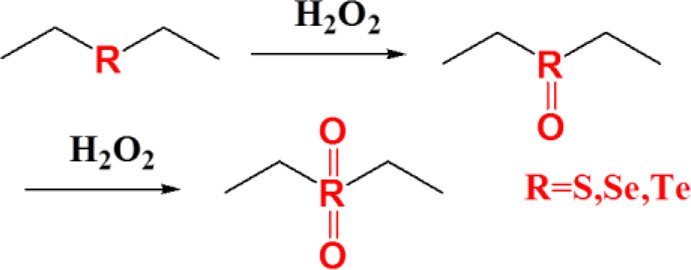
Structural cleavage		
Thioketal	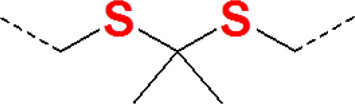	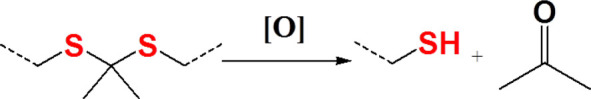
Arylboronic acid/esters containing polymers	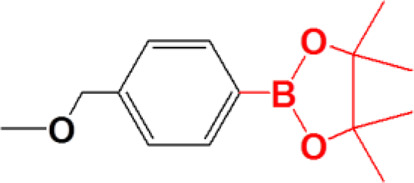	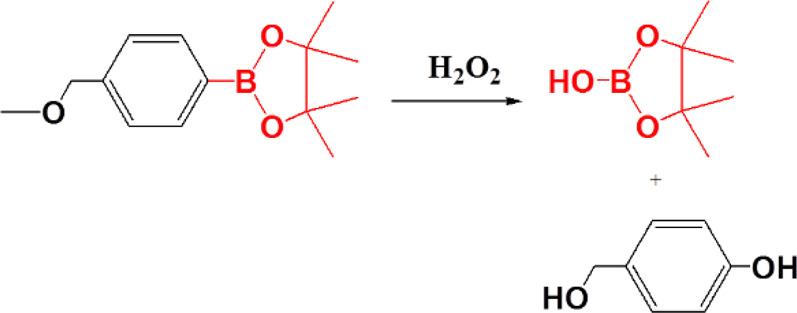
Aryl oxalate ester	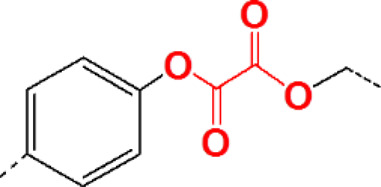	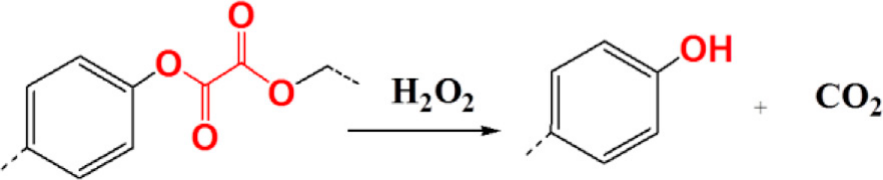
Proline oligomer	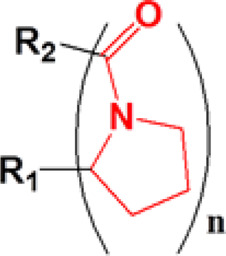	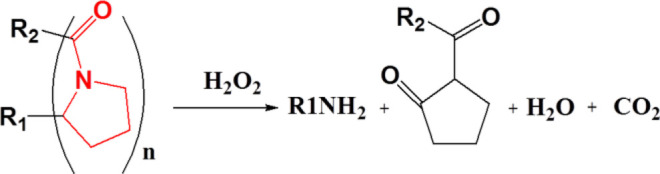
Ferrocene	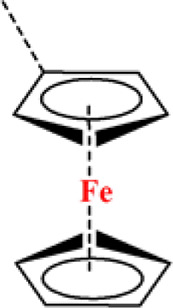	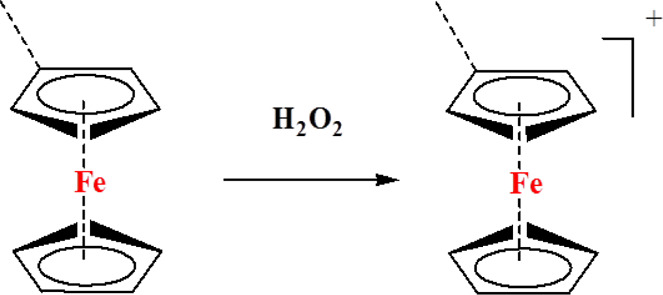
			
			

## Sulfur-Containing Polymers

Sulfur is a crucial and fundamental element for all living things. Many amino acids contain a sulfur group, such as cysteine, methionine, homocysteine, and taurine, and some universal enzymes also contained sulfur. Sulfur is an active element of the chalcogen family, which is readily available in nature. It shares two electrons with other elements, forming compounds with oxidation states of −2, +6, +4, +2, and +1. Sulfur is typically nontoxic, and consequently, sulfur-containing polymers for drug delivery have been extensively exploited over the past two decades, mostly because of the abundant polymer structure, the low toxicity, and their biocompatibility.

### Thioether-Containing Polymers

Thioether-containing polymers are one of the most investigated ROS-responsive materials. Thioethers are characterized by phase transition from hydrophobic to hydrophilic induced by ROS. Thioether groups are easily converted into sulfoxide under mild conditions in the presence of oxidative factors at low temperature and may be turned into sulfones under strong oxidative conditions. This oxidation reaction process changes the solubility of the polymer and leads to the decomposition of the nanocarriers and, subsequently, release of the payload (Hu and Tirelli, 2012). Thus, thioether-containing polymer phase transition induced by ROS is crucial for targeted drug release. Poly(propylene sulfide) (PPS) is one of the most interesting thioether-containing polymers. It had been extensively studied over the past two decades on account of its specific properties. PPS had a suitable *T*_*g*_ and hydrophobic performance, which easily converts into hydrophilic under the microenvironment of the lesion in the presence of elevated ROS. Moreover, OCl^¯^ oxidizes PPS much faster than H_2_O_2_. PPS is mainly oxidized into poly(propylene sulfoxide) by H_2_O_2_, while more sulfone groups are produced following treatment with OCl^¯^. In a pioneering study on PPS for drug delivery, Hubbell et al. synthesized an amphiphilic copolymer using poly(ethylene glycol) (PEG) as the hydrophilic block and the PPS as the hydrophobic block. Owing to low *T*_*g*_ and oxidative phase conversion, the amphiphilic copolymer vesicles could be further oxidized and destabilized (Napoli et al., 2004). Subsequently, various polymers containing thioether groups composed the main backbone, and the side chains or the tail chains have been developed and synthesized for application in biomedicine and biotherapy for cancer, neurodegenerative diseases, and inflammatory disorders (Yang et al., 2020).

**FIGURE 2:**
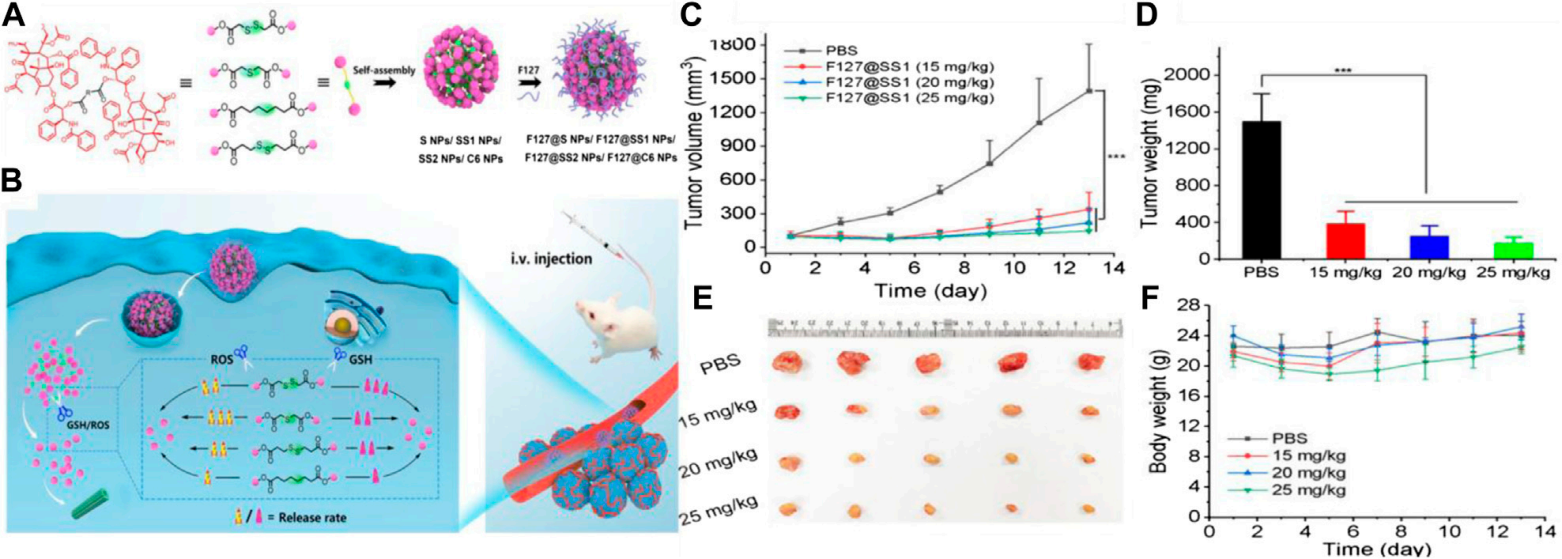
**(A)** Synthetic routes of the four types of PTX dimer prodrug nanoparticles. **(B)** Schematic diagram of ROS- and GSH-responsiveness of nanoparticles in tumor cells. **(C)** Curves of tumor volume treated with different prodrug nanoparticles, respectively. **(D)** Changes in tumor weights following treatment with different prodrug nanoparticles. **(E)** Photographs of excised tumors. **(F)** Body weight of tumor-bearing mice (Wang et al., 2011). Copyright 2020, the American Chemical Society.

Polymers containing thioether in the main chain have been developed mainly using transfer polymerization, ring-opening polymerization (ROP), and step-growth polymerization. For instance, Walker et al. explored nanoparticles (< 150 nm) composed of PPS-containing thioether inserted in its backbone by ROP (Allen et al., 2011). The nanoparticles were able to undergo phase transition from hydrophobic to hydrophilic, because the polysulfide was transformed into polysulfoxides and polysulfones under ppm levels of NaOCl. The rate of drug release under the oxidizing agent was evaluated, and the authors found that the nanoparticle formation was triggered in the presence of low concentrations of NaOCl (200 mM) and H_2_O_2_ (500 μM). These nanoparticles containing thioether in the main chain were promising constructs for controlled encapsulation and release of a variety of hydrophobic drugs. A hydrophobic anticancer drug 7-ethyl-10-hydroxyl-camptothecin (SN38) and a hydrophilic oligomer of ethylene oxide oligo (ethylene glycol) (OEG) were produced using the thioether chain connection (OEG-2S-SN38). The drug loading rate of OEG-2S-SN38 nanocapsules could achieve 35 wt%. The nanocapsules quickly collapsed, and more than 80% of the SN38 loaded was released within 15 min in the presence of 5 mM H_2_O_2_. The ROS sensitivity indicated that polymers containing thioether in the main backbone could act as an ideal drug delivery carrier (Wang et al., 2013). Lo et al. developed ROS and glutathione (GSH) dual redox-responsive micelles, which underwent thioether bond oxidation in the presence of high ROS levels, and the disulfide structure was cleaved in the presence of GSH (Chiang et al., 2015). Hu et al. also systematically studied the sensitivity of thioether and disulfide structure under higher expression of GSH and ROS in cancer cells. Four types of paclitaxel (PTX) dimers with different linkers were synthesized through esterification, as shown in Figure 2. The author’s results indicated that the thioether bond had a higher ROS sensitivity because of the lower negative potential, and the thioether bond could be readily oxidized in the presence of 10 mM H_2_O_2_ in comparison to the disulfide bond. Conversely, the disulfide bond exhibited higher GSH sensitivity due to the presence of –SH, and an exchange reaction occurred. The study provided a design rationale for ROS and GSH dual-responsive prodrugs or carriers using thioether bond and disulfide bonds, which underlined the significance of ROS polymers containing the thioether backbone structure (Wang et al., 2011).

**FIGURE 3:**
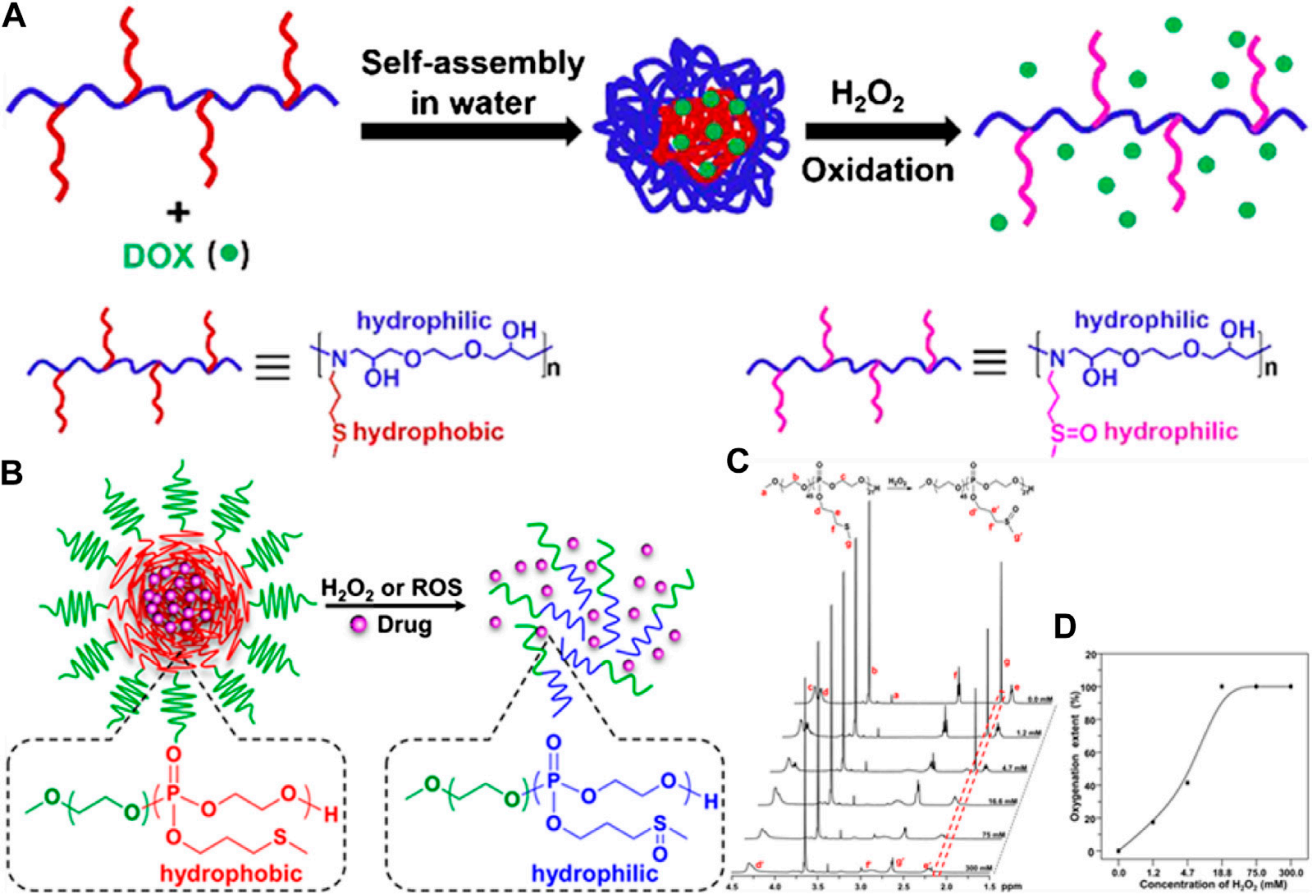
**(A)** The chemical structure of thioether side chain polymer carriers and the drug release behavior of DOX-loaded P(MSPA-α-EG) micelles (Wang et al., 2019). Copyright 2019, the American Chemical Society. **(B)** Schematic illustration of thiother-pendant monomers MSPEP for polymerization of ROS-responsive carriers **(C)** 1H NMR spectra of mPEG-b-PMSPEP carried out following oxidation in the presence of different H_2_O_2_ concentrations. **(D)** Oxygenation degree of mPEG-b-PMSPEP in the presence of different H_2_O_2_ concentrations (Wang et al., 2019). Copyright 2019, the American Chemical Society.

Polymers with thioether side groups could present more diversified structures by the introduction of a variety of thioether monomers, such as 3-(methylthio)-propylamine (MSPA) (Wang G. C. et al., 2019), 2-(methylthio)-ethyl glycidyl ether, and 3-(methylthio)propyl ethylene phosphate (MSPEP) (Wang J. H. et al., 2019). When the thioether in the side chains reacts with ROS, the polymer’s main chain is not damaged and maintains the structure of the polymer’s backbone chain. Therefore, thioether-containing polymers in side chains were often used as multifunctional drug carriers. Huang et al. synthesized an amphiphilic polymer (P(MSPA-α-EG)) containing thioether in the side chains through an amine–epoxy click reaction at room temperature. The anticancer drug doxorubicin (DOX) could be encapsulated by P(MSPA-α-EG) micelles with a diameter of approximately 151 nm, and the DOX load and loading efficiency were 4.90 and 9.81%, respectively. DOX could be released rapidly from DOX-loaded P(MSPA-α-EG) micelles in the presence of 20 mM H_2_O_2_, and the cumulative release of DOX could reach up to 55.6%. The chemical structure of polymer carriers and the drug-releasing behavior of the DOX-loaded P(MSPA-α-EG) micelles are shown in Figure 3A (Wang et al., 2019). Wang et al. synthesized an amphiphilic copolymer (mPEG-b-PMSPEP) with thioether side groups using ROP. The photosensitizer chlorin e6 (Ce6) and anticancer drug PTX were successfully packaged into mPEG-b-PMSPEP nanoparticles. The mPEG-b-PMSPEP nanoparticles were responsive to ROS generated by light and a photosensitizer. Further, the encapsulated mPEG-b-PMSPEP nanoparticles underwent oxidation in the presence of ROS generated by light irradiation, achieving an irradiation-accelerated PTX release. The chemical structure of mPEG-b-PMSPEP and the encapsulated mPEG-b-PMSPEP nanoparticles are shown in Figure 3B; Wang et al., 2019. In addition, the drug carrier can be modified to contain thioethers in polypeptide side chains (Fu et al., 2014).

**FIGURE 4:**
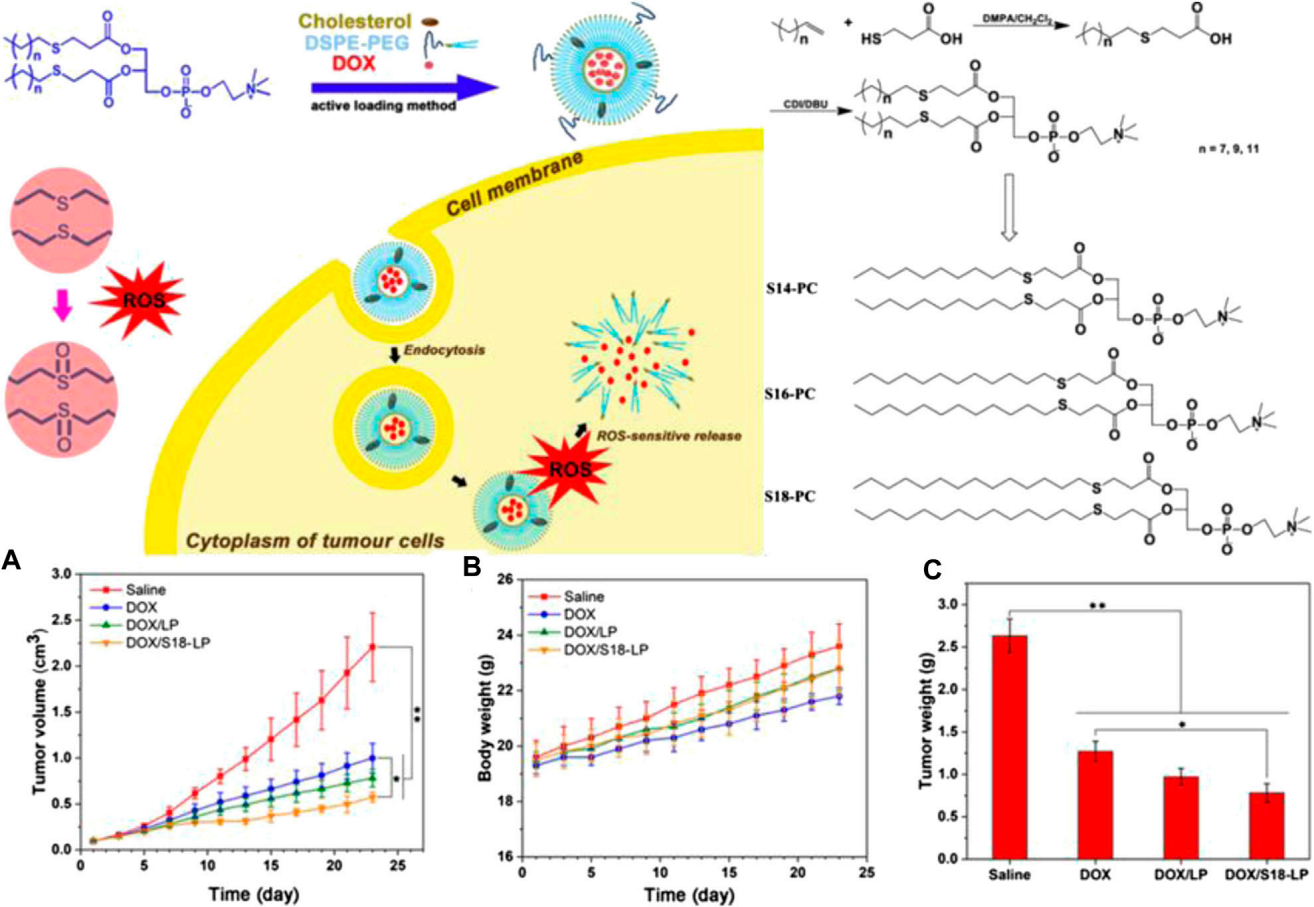
Illustration of ROS-responsive polymers containing thioether in the tail chains and the resulting chemical structure. **(A)** Curves of tumor volume treated with different S-PC–based liposomes. **(B)** Body weight of tumor-bearing mice. **(C)** Curves of tumor weight treated with different S-PC–based liposomes (Du et al., 2019). Copyright 2019, the American Chemical Society.

Polymers containing thioether in the tail chains have also been developed. The chain length of polymer tails is relatively easy to control when the thioether linker is used in end-capping the polymer terminal. This construct also maintains the backbone function intact as well as the thioether in the side chain. Li et al. synthesized S14-COOH, S16-COOH, and S18-COOH using olefin and thiohydracrylic acid using the click reaction as the first step (Figure 4). The second step involved the preparation of three thioether-containing phosphatidylcholines (S-PCs) with different tails, and S14-COOH, S16-COOH, and S18-COOH were used as end-capping agents by esterification reactions. The phase transition temperature and self-assembly ability were determined by the structures of the different length of the tail chains for the S-PCs. Further, S-PC–based liposomes (S-LPs) have been used for ROS-responsive controlled release of DOX. The testing of the anticancer effects including changes in tumor volume, body weight, and tumor weight *in vivo* showed that the polymer containing thioether in the tail chains had tremendous potential as a ROS-responsive polymer drug delivery construct for tumor therapy (Du et al., 2019).

**FIGURE 5:**
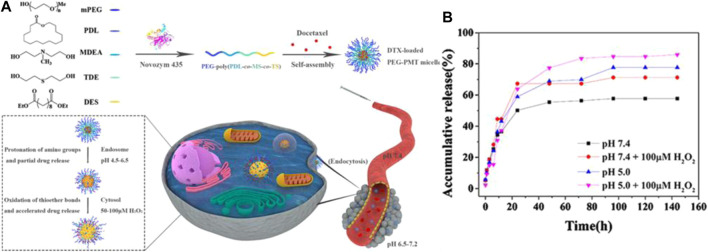
**(A)** The preparation of PEG-PMT nanoparticles and schematic diagram of ROS/PH responsive micelles for DTX delivery in tumor cells. **(B)** Cumulative release of DOX from DTX-loaded PEG-PMT micelles in the presence of the different pH (5.0 and 7.4)100 μM H_2_O_2_ at different time (Su et al., 2020). Copyright 2020, Elsevier.

Various multiple-responsive systems can be achieved when thioether groups are combined with other stimuli-responsive functional groups, such as ROS/temperature (Xiao et al., 2015), ROS/PH (Su et al., 2020), ROS/light, and GSH/ROS (Yin et al., 2020). For example, Chen et al. developed ROS/temperature dual-responsive polymer carriers (PEG–EDT) *via* thiolene polymerization of poly(ethylene glycol)diacrylate (PEGDA) and 1,2-ethanedithiol (EDT) monomers. PEG–EDT exhibited a reversible temperature-induced phase transition due to, obviously, synergy between the hydrophobic interaction of the thioether and the dehydration of PEG. Additionally, the PEG–EDT copolymers also possessed ROS-responsive behavior due to the presence of the thioether groups in the backbone chain (Xiao et al., 2015). Liu et al. synthesized pH/ROS dual-responsive biodegradable polymer carriers (PEG-PMT) *via* lipase-catalyzed copolymerization. The PEG-PMT nanoparticle sizes increased sharply in the presence of oxidative factors and the acidic environments due to the contribution of protonation of the thioether groups and the oxidation of the amino groups. DOX could be released rapidly from DTX-loaded PEG-PMT micelles at pH 5.0 and in the presence of 100 μM H_2_O_2_ after 144 h. The cumulative release of DOX, over 85% of the anticancer drug load, is shown in Figure 5B; Su et al., 2020). These experimental results demonstrated that multiple-responsive polymer carriers can be modified by functional groups to ensure more efficient controlled release of drug dosage forms for targeted drug delivery systems than single-responsive polymer carriers.

Taken together, thioether-containing polymers provide a compelling approach for loading hydrophobic cytotoxic drugs and improving the efficiency of targeted drug delivery for the treatment of cancer and inflammatory diseases. Meanwhile, exploiting different structural designs of thioether-containing polymers will likely have strong application potential.

### Poly(thioketal)

Unlike the thioether-containing polymer described above, the application of poly(thioketal) exploits the characteristic thioketal (TK) bond that can be cleaved following ROS induction to produce acetone and two other thiol-containing fragments, which then lead to polymer chain scission and breakdown. The chemical structure of TK is very similar to that of the thioether group (see Table 1 for details). Previous studies have shown that TK undergoes oxidative reactions with several kinds of ROS, including H_2_O_2_, •OH, and O_2_•¯ (Shim and Xia, 2013), which implies that the TK bond could be used as the functional group in ROS-responsive polymers for drug delivery. More importantly, the by-product of TK cleavage could be metabolized easily (Zhang Y. et al., 2017). Therefore, TK is an important component for the design of ROS-responsive polymers.

**FIGURE 6:**
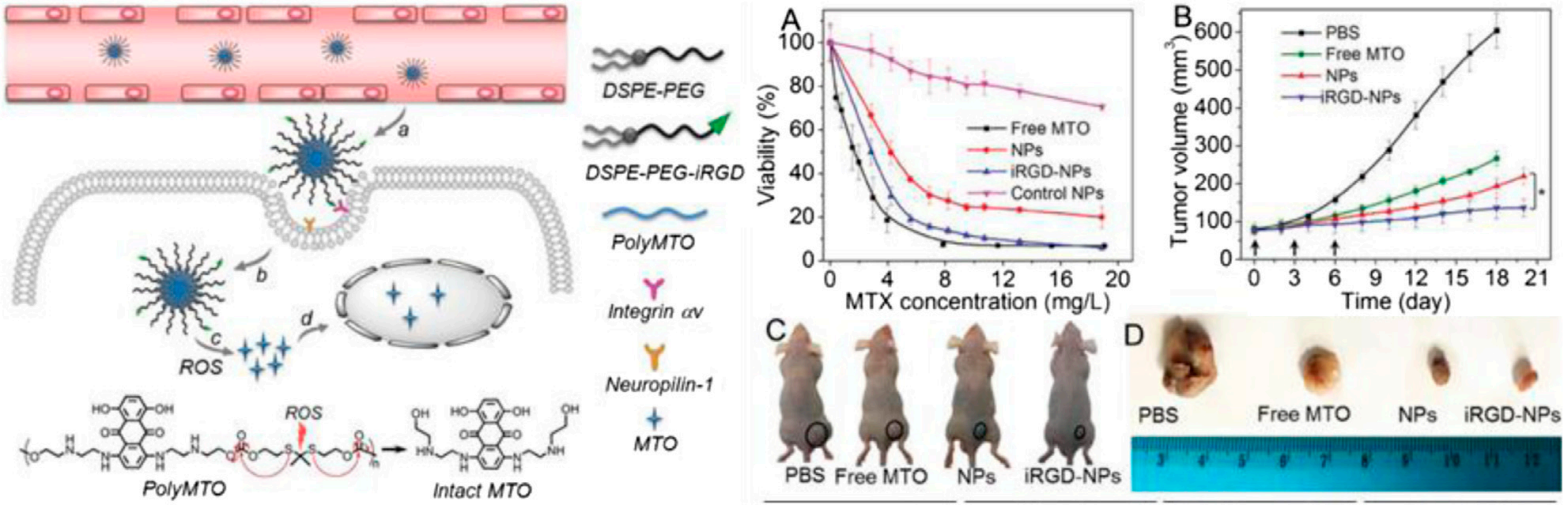
Schematic diagram of the mechanism for release of intact anticancer drug molecules mitoxantrone (MTO) and the chemical structure of polyMTO. **(A)** Cytotoxic effects induced by different polyMTO-based nanoparticles. **(B)** Curves of tumor volumes following treatment with different prodrug polyMTO-based nanoparticles. **(C)** Photograph of tumor-bearing mice; tumors are indicated by circles. **(D)** Photograph of tumor volume after treatment with PBS and various prodrug polyMTO-based nanoparticles (Xu X. et al., 2017). Copyright 2017, John Wiley and Sons.

TK-containing polymers in the main chain can be synthesized by direct condensation polymerization using thiols (Natalia et al., 2019). Xu et al. prepared a polyprodrug (polyMTO) with a ROS-responsive TK group by condensation polymerization for targeted cancer therapy. PolyMTO nanoparticles had the advantages of high drug loading, longer blood circulation, targeted drug delivery, and release of intact anticancer drug molecules such as mitoxantrone (MTO). These advantages were attributed to the ability of TK-containing linkage to be cleaved in the presence of ROS. The experimental data *in vivo* showed that the polyMTO-based nanoparticles had significant therapeutic effects on inhibiting tumor cell growth (see Figure 6B for details; Xu X. et al., 2017). He et al. also synthesized a poly(ester-thioacetal) copolymer and introduced the functional group thioacetal and cinnamaldehyde (CA) by condensation polymerization. When the thioacetal bond cleaved in the tumor microenvironment, the CA from the polymer chain was released to prompt mitochondria to regenerate ROS. The process synergistically increased the anticancer treatment efficiency and achieved high-efficiency drug delivery (Xu et al., 2018a).

**FIGURE 7:**
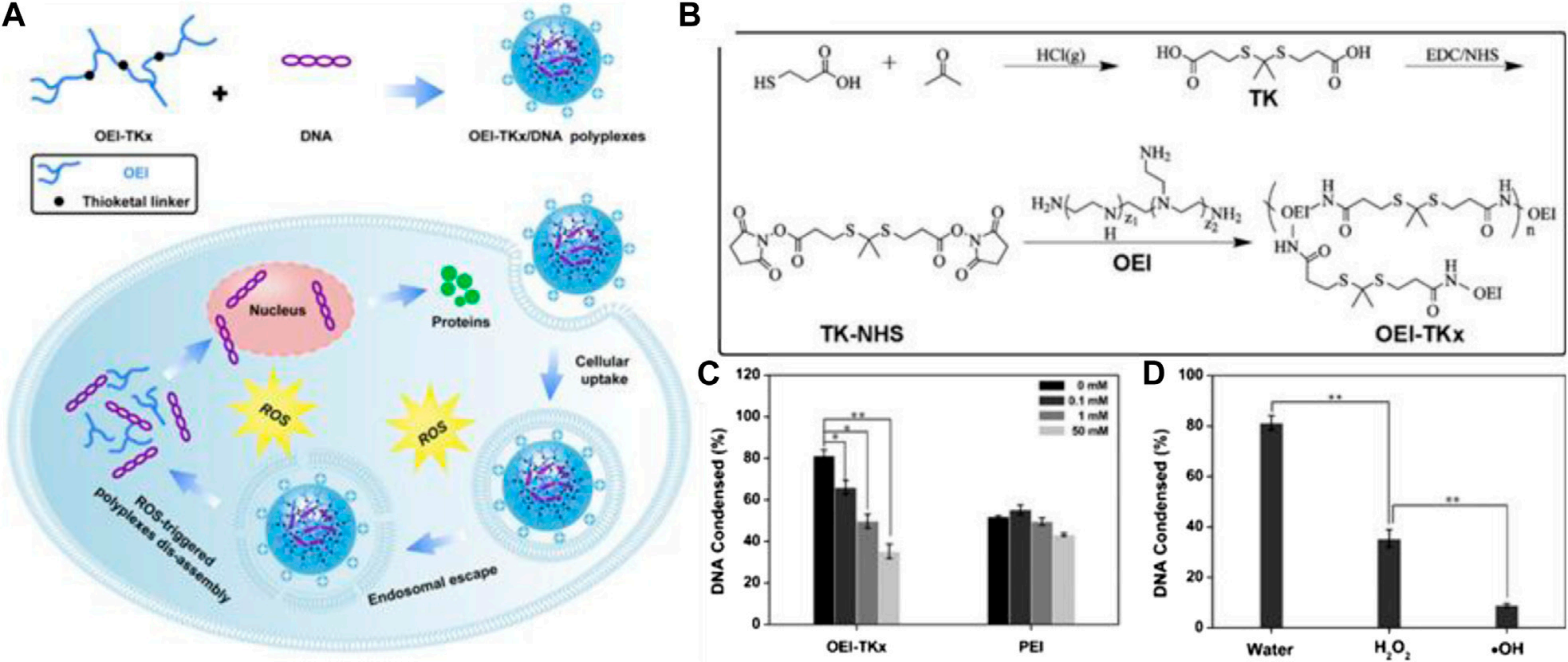
**(A)** Schematic diagram of OEI-TKx/DNA polyplexes for gene delivery. **(B)** Synthetic routes of the TK-containing cross-linkers and OEI-TKx. **(C)** Percentage of DNA condensation with different H_2_O_2_ concentrations; the gene release efficiency of OEI-TKx/DNA polyplexes was determined by the EtBr exclusion assay. **(D)** Graph of ROS-responsive DNA release from OEI-TKx/DNA under various conditions (Zhang et al., 2019). Copyright 2019, the American Chemical Society.

TK-containing polymers in the side chain were synthesized by various TK-containing cross-linkers instead of direct condensation polymerization using thiols. Two main reasons explain why monomers containing thiols were easily oxidized and why the condensation polymerization method was often unable to prepare high molecular weight and narrow the polydispersity of polymers. This method that is often adopted involves various TK-containing cross-linkers to be synthesized and then used to synthesized TK-containing polymers. The advantage of the TK cross-linker is that it prolongs blood circulation time due to improvement of stability and accelerates drug release in response to the excess ROS found in the tumor microenvironment (Zhang Y. et al., 2017). Gu et al. synthesized TK-containing cross-linkers using 3-mercaptopropionic acid and acetone as the first step, and then a water-soluble polymer (OEI-TKx) was synthesized *via* TK cross-linking. The synthetic route is shown in Figure 7B. The OEI-TKx degradation performance was investigated upon exposure to 100 mM H_2_O_2_ containing 1.6 μM CuCl_2_ at 37°C. The molecular weight dropped significantly, and the level of degradation was consistent with that of oligoethylenimine (OEI) without TK following simultaneous exposure to 400 mM H_2_O_2_ containing 1.6 μM CuCl_2_. The results demonstrated that OEI-TKx had the ability to respond to ROS, in which the TK group played an important role. The OEI-TKx/DNA polyplexes were also developed to produce higher gene release efficiency. The gene release efficiency of OEI-TKx/DNA polyplexes was detected using the ethidium bromide (EtBr) exclusion assay, whereby the percentage of DNA condensation decreased with the sharp increase in H_2_O_2_ concentration, as shown in Figure 7C. Thus, the unique properties of TK linkages may have potential application as stimulus-responsive materials for gene delivery (Zhang et al., 2019).

**FIGURE 8:**
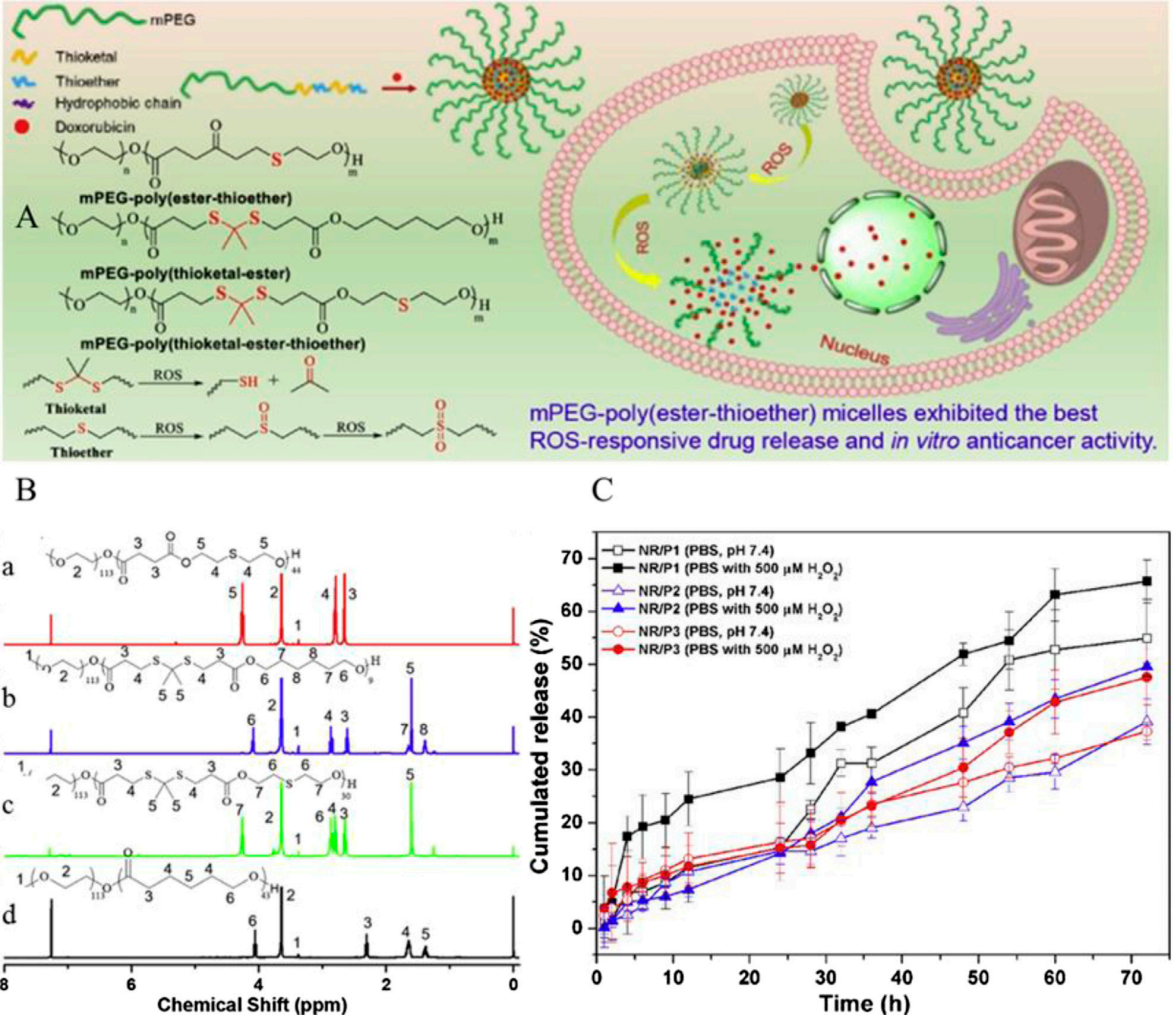
**(A)** Schematic diagram of ROS-responsive nanoparticles and chemical structure of mPEG–poly(ester-thioether), mPEG–poly(thioketal-ester), and mPEG–poly(thioketal-ester-thioether). **(B)** Structures of mPEG–poly(ester-thioether) **(a)**, mPEG–poly(thioketal-ester) **(b)**, mPEG–poly(thioketal-ester-thioether) **(c)**, and mPEG-b-PCL **(d)** were confirmed by 1H NMR spectra using CDCl_3_ as the solvent. **(C)** Graph of the DOX-loaded P1, P2, and P3 micelle drug release in 500 μM H_2_O_2_ (Xu et al., 2019). Copyright 2019, Elsevier.

Furthermore, TKs in combination with thioether have also been developed for drug delivery. Poly(thioketal) and thioether-containing polymers are both sulfur-containing ROS-responsive polymer carriers, but as mentioned above, they exhibit completely different ROS-responsive mechanisms. If the TK bonds and thioether bonds exist together linked by covalent bonds on the same polymer chain as functional groups, the polymer may have an enormous effect on drug release. Xu et al. synthesized an amphiphilic copolymer micelle mPEG–poly(ester-thioether) (P1) using thiodiglycol (TDG) and methoxy poly(ethylene glycol) (mPEG), mPEG–poly(thioketal-ester) (P2), and mPEG–poly(thioketal-ester-thioether) (P3). Similar to P1, mPEG-b-PCL (P4) had also been synthesized in order to compare the properties of the three ROS-responsive polymers. The chemical structures and the 1H NMR spectra of the copolymers are presented in Figure 8. The drug loading capacity of the DOX-loaded P1, P2, P3, and P4 micelles was evaluated, and the three ROS-responsive polymer micelles all exhibited better drug loading efficiency than P4 micelles without ROS sensitivity. P3 micelles presented the best drug loading efficiency with drug loading content and the encapsulation efficiencies of 13.7 and 68.35%, respectively. The drug release of the DOX-loaded P1, P2, and P3 micelles was inspected in the presence of 500 μM H_2_O_2_ (Figure 8C). The DOX-loaded P1 micelle exhibited the fastest drug release rate, and the cumulative release rates reached to 65% (Xu et al., 2019). Furthermore, TK bonds were stable under acidic, alkali, or protease conditions. Therefore, TK-containing polymers were optimal for therapeutics targeting inflamed tissues in the intestine (Wilson et al., 2010).

## Selenium-Containing Polymers

Selenium is an essential element in the human body and plays a significant role in protecting cells from oxidative damage. The lack of selenium in the human body may lead to Keshan disease and depression. Selenium has a higher atomic number and lower electronegativity than sulfur; thus, selenium-containing polymers have lower bond energy than sulfur-containing polymers (C–Se bond 244 kJ/mol, Se–Se bond 172 kJ/mol, C–S 272 kJ/mol, and S–S 240 kJ/mol). Selenium-containing polymers are a promising biomaterial for targeted drug delivery due to their more sensitive response to ROS. Selenide can be oxidized to selenoxides and selenones, having a mechanism of action similar to thioether, and oxidation leads to phase transition from hydrophobic to hydrophilic. Diselenide bonds can be oxidized to seleninic acid and then are reduced to selenol and ultimately cracked, which is a mechanism of action that is similar to that of disulfide bonds. The only difference is that selenium-containing polymers possess higher sensitivity to oxidants than sulfur-containing polymers (Xu et al., 2013). Therefore, selenium-containing polymers are more widely used in environments with lower ROS concentrations (Liu et al., 2012).

The interest in the synthesis of selenium-containing polymers had been limited for a long time, as selenide and diselenide bonds were unstable in the presence of oxygen and given the poor solubility of the polymer. This view changed in 2010, when a groundbreaking study by Xu and Zhang et al. (Ma et al., 2010b) discovered that solubility could be increased by introducing the diselenide group into a diol structure. Amphiphilic diselenide-containing polyurethane (PUSeSe) was synthesized using toluene diisocyanate (TDI) and diselenide-containing diols *via* stepwise polymerization. Since then, many copolymers have incorporated selenium-containing polymers and have resulted in the synthesis of ROS-responsive polymer carriers. Sun et al. developed a series of selenium-containing polymers by coupling reactions such as the amphiphilic di-block polymer (MPEG–IPDI–Se–Se–IPDI–PPG), which was synthesized using PEG, di(1-hydroxyethylene) diselenide, monomethyl ether (mPEG), and polypropylene glycol (PPG) *via* four coupling reactions (Sun et al., 2013a) and an amphiphilic triblock polymer (Se–Se–tri-ABP) (Sun et al., 2013b). Selenium-containing macrocyclic monomers have been developed to synthesize selenium-containing polymers as the backbone *via* living ring-opening polymerization (ROP) in the presence of lipase CA (Liu T.-I. et al., 2018; Wei et al., 2018). Subsequently, Xu and Zhang et al. developed a series of ROS-responsive selenium-containing polymers for drug delivery. According to the position of the selenide and diselenide bonds in the polymer chain, the polymer could be divided into polymers containing selenium in the main chain (Ma et al., 2010a; Tan et al., 2012; Xia et al., 2017; Zhou et al., 2017), in the side chain (Han et al., 2010; Ren et al., 2012), and in dendritic selenium-containing polymers (Xu et al., 2006; Fu et al., 2012; Li et al., 2016).

**FIGURE 9:**
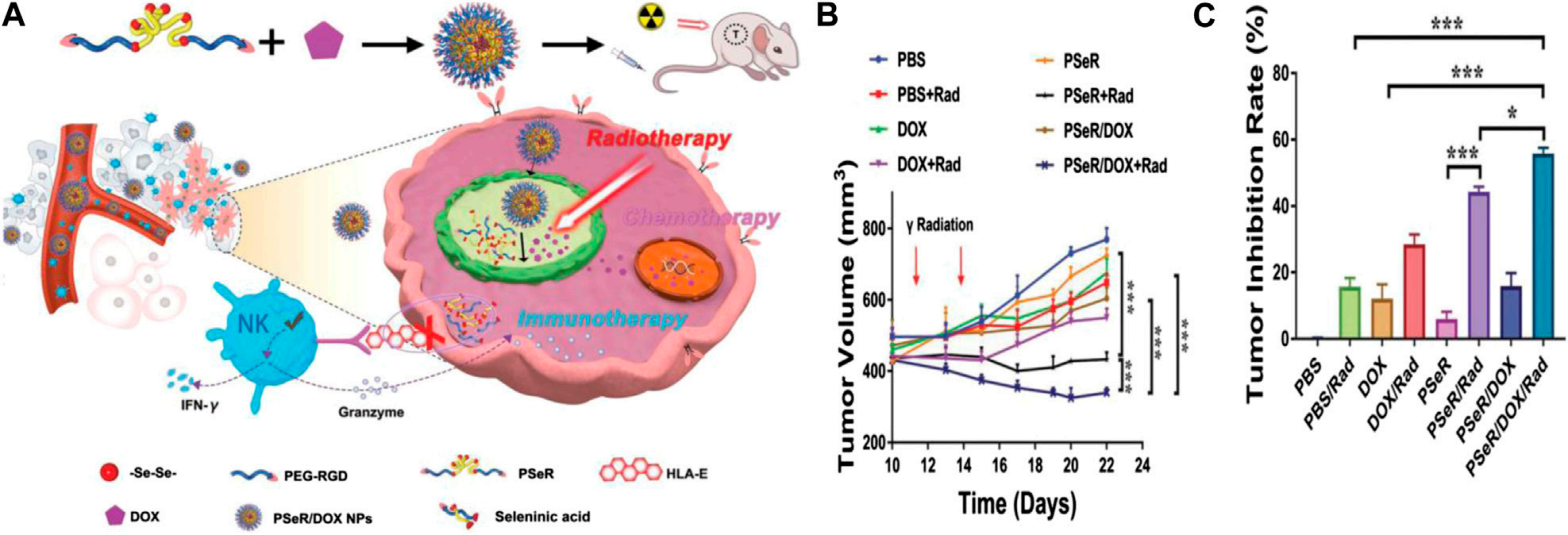
**(A)** Schematic illustration of the PseR/DOX mechanism of therapeutic action and chemical structure. **(B)** Curves of tumor volumes following treatment with different PseR-based nanoparticles. **(C)** Curves of tumor inhibition rates following treatment with different PseR-based nanoparticles (Gao et al., 2020). Copyright 2020, John Wiley and Sons.

In addition, to reduce side effects, selenium-containing polymers were adopted for application in ROS- and light-responsive drug delivery systems for chemotherapy and radiotherapy (Li T. Y. et al., 2020). The light sources include red light (Han et al., 2013), visible light (Ren et al., 2013; Sun et al., 2017), and γ-radiation (Ma et al., 2011). Recently, Xu et al. synthesized PSeR/DOX, a diselenide-containing polymer (PseR), using 11, 11′-diselanediylbis-(undecan-l-ol) (DseOH), RGD, and PEG through stepwise polymerization. PseR nanoparticles could be oxidized to seleninic acid when treated with 5 and 2.5 Gy (1 Gy min^−1^) γ-ray radiation, which were much weaker levels than the normal cancer therapy doses given *in vivo*. The release of DOX was greatly improved as ROS and γ-radiation exerted a synergistic effect during this chemical process. The PseR/DOX formulation could effectively inhibit the tumor growth, tumor volume, and growth rates (Figure 9; Gao et al., 2020). The preparation had high application potential to achieve radiotherapy, chemotherapy, and immunotherapy simultaneously, and thus, selenium-containing polymers have also been used as ROS-responsive polymer carriers because of their high sensitivity for ROS in selenium-containing polymers (Xia et al., 2018; Sun et al., 2020).

## Tellurium-Containing Polymers

Tellurium is another important element in the chalcogen family and is an unnecessary and potentially toxic element in human body. Tellurium is very rare in nature partly due to its high atomic number. Tellurium-containing polymers possess higher sensitivity to ROS than selenium-containing polymers. Tellurium-containing polymers may find potential application as ROS-eliminating materials, and not as agents for ROS-responsive delivery of drugs under physiological environment (Cao et al., 2014; Wang et al., 2020).

The synthesis of polymers containing tellurium is similar to that of polymers containing selenium. Xu et al. have carried out most of the related research on tellurium-containing polymers. The group has reported that co-assembly *via* tellurium-containing molecules and phospholipid exhibits ultrasensitive ROS-responsive properties and biocompatibility. The co-assemblies respond to the ROS concentration present in physiological conditions (100 μM H_2_O_2_) due to tellurium (Wang et al., 2015). Linear tellurium-containing polymers (PEG-PUTe-PEG) are responsive to 100 μM H_2_O_2_ and can be coupled with radiation therapy. The ROS produced by γ-ray radiation (2 Gy) may trigger phase transition of the PEG-PUTe-PEG (Cao et al., 2015). Tellurium-containing hyperbranched polymers have also been synthesized, and the influence of the degree of cross-linking degree on the ROS-responsive behavior was investigated. Hyperbranched polymer-containing tellurium swell upon exposure to ROS concentrations in physiological conditions (100 μM H_2_O_2_) in an aqueous environment, and this property could be used to eliminate excess ROS levels (Fang et al., 2015).

**FIGURE 10:**
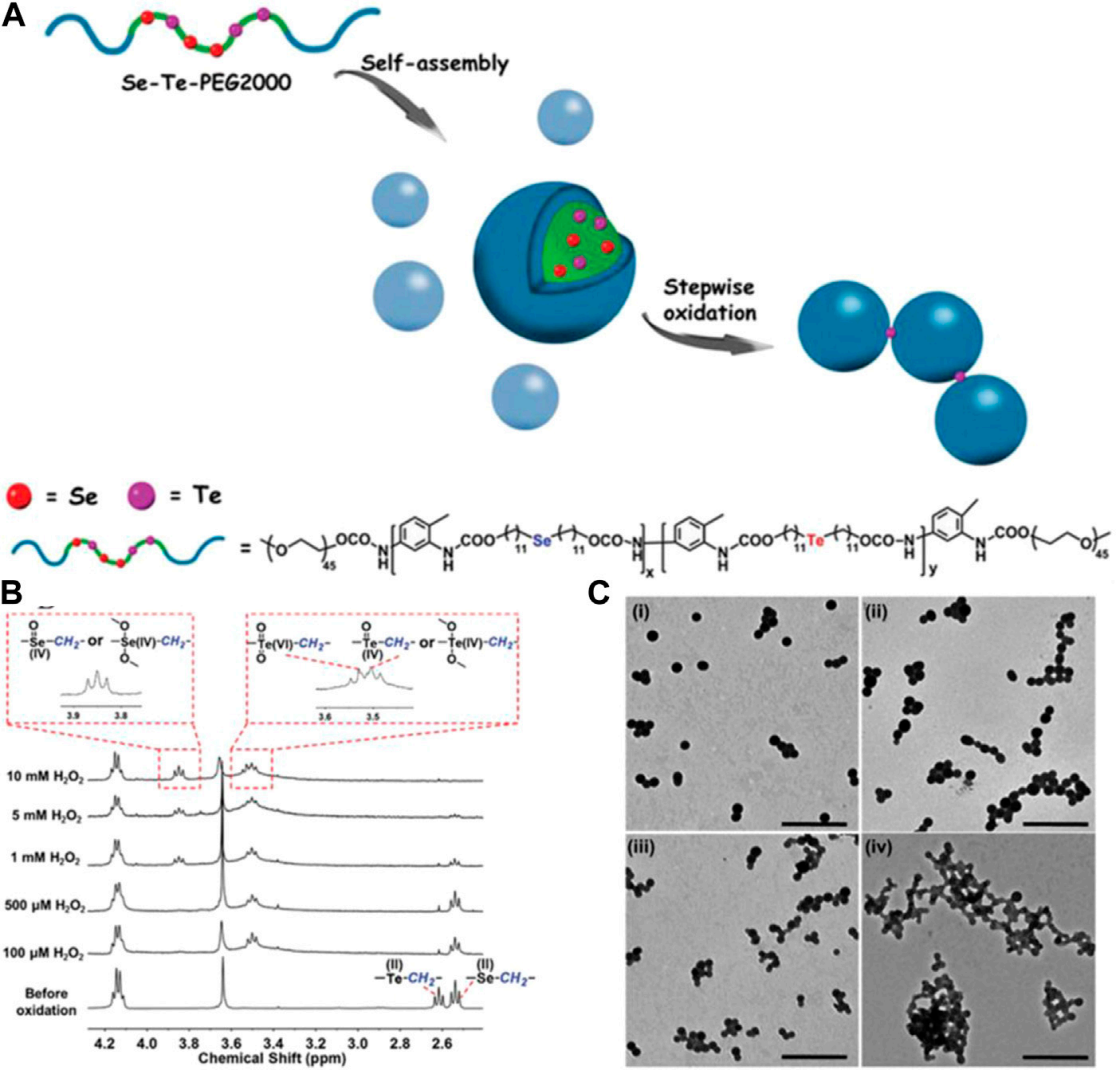
**(A)** Schematic illustration of Se–Te–PEG2000 mechanism of stepwise oxidized activity and chemical structure. **(B)** 1H NMR spectra of Se–Te–PEG2000 before and after oxidation for 5 h when exposed to different concentrations of H_2_O_2_. **(C)** TEM images of Se–Te–PEG2000 after oxidation by 10 mM H_2_O_2_ for different oxidation periods: **(i)** 5 h, **(ii)** 12 h, **(iii)** 24 h, and **(iv)** 72 h (Wang et al., 2017). Copyright 2017, the Royal Society of Chemistry.

In addition, an amphiphilic multi-hierarchical responsive polymer (Se–Te–PEG2000) comprising the hydrophobic blocks selenium- and tellurium-containing polyurethane and the hydrophilic blocks PEG mono-methyl ethers has also been designed and synthesized. Se–Te–PEG2000 was synthesized using di-(1-hydroxylundecyl) selenide (MseOH), TDI, and di-(1-hydroxylundecyl) telluride (MteOH). The chemical structure and the 1H NMR spectra of Se–Te–PEG2000 are shown in Figure 10. Hierarchical responsive properties of Se–Te–PEG2000 were derived from the selenium, while tellurium had different sensitivities to oxidation. Thus, Se–Te–PEG2000 could undergo stepwise oxidization by tuning the concentration of the oxidant (chemical methods) or by tuning the voltage during exposure to electrochemical stimuli (electrochemical methods) (Wang et al., 2017).

## Arylboronic Acid/Ester-Containing Polymers

Among the various functional groups of ROS-responsive reaction, arylboronic acid and ester are unique because they are highly selectively to oxidation by H_2_O_2_ and generate phenol and boronic acid as the oxidation products (Li et al., 2017). This feature has enabled to explore ROS-responsive targeted drug delivery polymers and has also received increasing attention (Deng et al., 2016; Stubelius et al., 2019).

**FIGURE 11:**
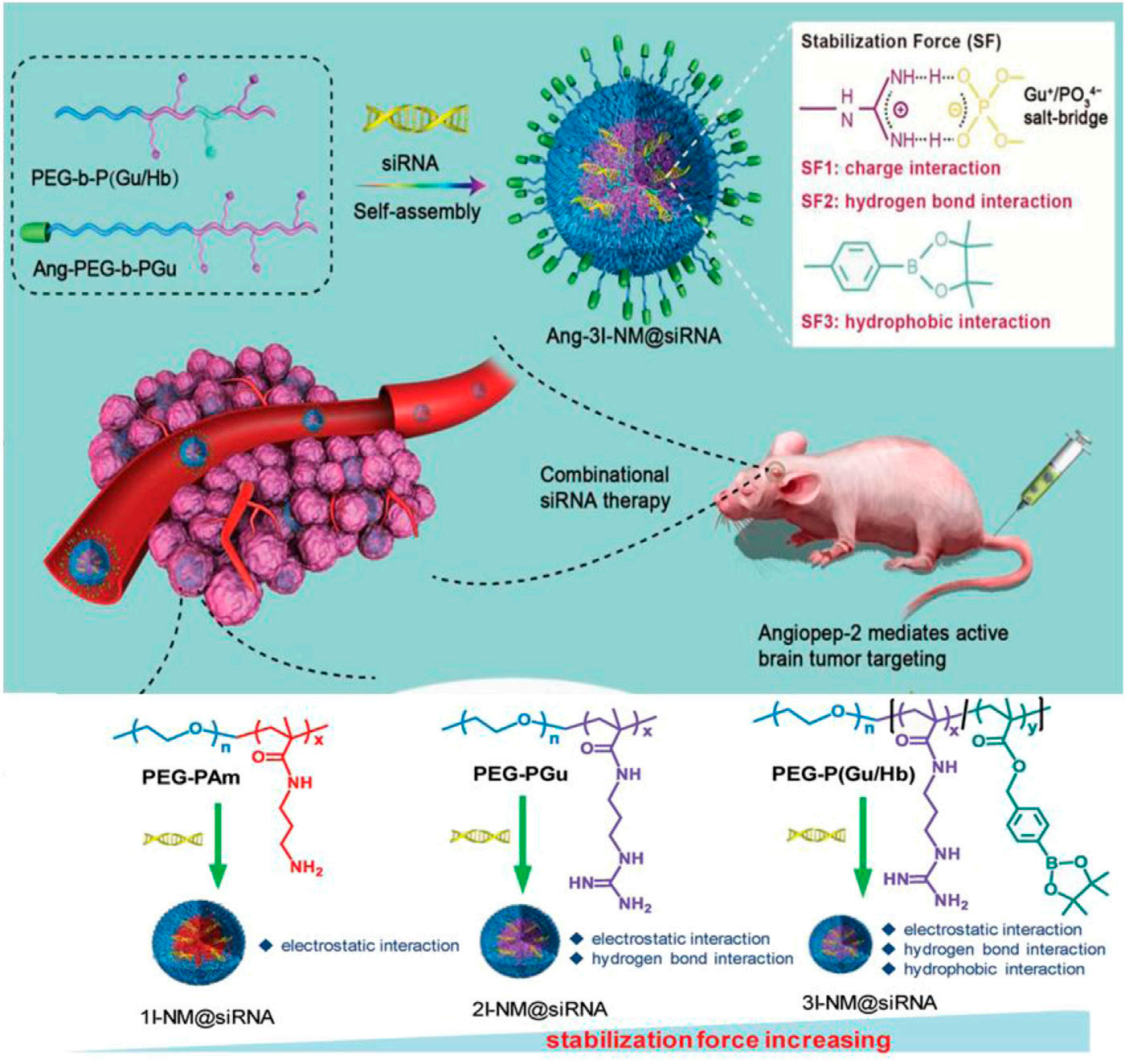
Schematic illustration of 3I-NM@siRNA stabilized by electrostatic, hydrogen bond and hydrophobic interactions. The chemical structures of poly(ethylene glycol)-blockpoly (N-(3-aminopropyl methacrylamide) (PEG-Pam), poly(ethylene-glycol)-block-poly(N-(3-methacrylamidopropyl) guanidinium) (PEG-b-PGu), and PEG-B-P (Gu/Hb) (Zheng et al., 2019). Copyright 2019, John Wiley and Sons.

Arylboronic acid/ester-containing polymers exhibit distinctive structures from those mentioned previously. Arylboronic acid/ester-containing structures rarely exist in the main backbone chain of a polymer, while polymers containing arylboronic acid/ester functional groups in the side chain have been developed by stepwise polymerization (Cui et al., 2017) and ROP (Qiu et al., 2017) with arylboronic acid/ester pendant monomers. Arylboronic acid/ester exhibits structural advantages that allow, specifically, conjugation to polyhydroxy compounds having an ortho- or meta-diol structure (Xin et al., 2019; Jager et al., 2020). Kuang et al. synthesized nitrogen mustard prodrugs starting from 4-(bromomethyl) phenylboronic acid pinacol ester. They were pioneers in the field and discovered that anticancer prodrugs could be activated by ROS to release DNA cross-linking agents (Kuang et al., 2011 19278–19281). Xin et al. developed a ROS-responsive neuroprotective drug delivery carrier (SHp-RBCNP) modified by a boronic ester group, stroke homing peptide (SHp), and a red blood cell (RBC). The SHp-RBCNP could prolong the systemic circulation of the neuroprotective drug (NR2B9C), enhance active targeting to the ischemic area in rat models of middle cerebral artery occlusion, and reduce ischemic brain damage (Lv et al., 2018). The dual-responsive arylboronic ester polymers were also synthesized as ROS/pH dual-responsive amphiphilic copolymers (Song et al., 2013). The design strategies of reactive arylboronic acid/ester nanodrugs have become increasingly sophisticated. Shi et al. reported a ROS-responsive polymer carrier (3I-NM@siRNA) constructed to carry small interfering RNA (siRNA) stabilized by electrostatic, hydrogen bond and hydrophobic interactions with the aim to improve siRNA circulation stability and delivery efficiency. The poly(ethylene glycol)-block-poly[(N-(3-methacrylamidopropyl) guanidinium-co-4-(4,4,5,5-tetramethyl-1,3,2-dioxaborolan-2-yl benzylacrylate)](PEG-B-P(Gu/Hb)) was prepared as shown in Figure 11. PEG-B-P(Gu/Hb) exhibits triple interactions, with the electrostatic and hydrogen bonding properties provided by the Gu^+^/PO_3_^4−^ bridge, and the arylboronic ester providing the hydrophobic interactions. Hydrophobic interactions were destroyed upon exposure concentrations of H_2_O_2_ 100 μM; however, when given in the presence of the arylboronic ester group, the preparation could respond to ROS, and the by-product carboxyl groups could subsequently interfere with electrostatic and hydrogen bond interactions. This complicated process resulted in the effective release of the siRNA nanomedicine (Zheng et al., 2019). Shen et al. also synthesized poly[(2-acryloyl)ethyl(p-boronic acid benzyl)diethylammonium bromide] (B-PDEAEA) for higher gene transfection efficiency. When B-PDEAEA is degraded by ROS, a negative charge reversal would cause the rapid release of DNA. In addition, boronate acid/esters and their ultimate products are considered nontoxic to humans (Liu et al., 2016).

## Other Polymer Systems

### Aryl Oxalate–Containing Polymers

Aryl oxalates may react with H_2_O_2_ to form 1,2-dioxetanediones, which can be rapidly converted into carbon dioxide (CO_2_) and phenols. Thus, aryl oxalate ester bonds in polymerized nanoparticles may induce degradation and then release the payload when exposed to H_2_O_2_ to achieve intelligent delivery of drugs (Kim et al., 2017).

Aryl oxalates as functional groups generally have been introduced in the backbone chain of the polymer or in the side chain. Polymers containing oxalate in the main chain can be synthesized by stepwise polymerization, and polymers containing oxalate in the side chain can be synthesized *via* ROP from oxalate-containing norbornene or fragmentation chain transfer (RAFT) polymerization reactions using oxalate-containing methacrylate. Aryl oxalates with negative groups are more beneficial to improve chemiluminescence than aliphatic oxalates. Polymers containing oxalate have been adopted to detect H_2_O_2_ using this property. Kang et al. synthesized a biodegradable p-hydroxybenzyl alcohol-incorporated copolyoxalate (HPOX) in a one-step condensation reaction of oxalyl chloride, 1,4-cyclohexanedimethanol, and p-hydroxybenzyl alcohol (HBA). HPOX degrades completely in the presence of H_2_O_2_, and the degradation products are cyclohexanedimethanol, HBA, and CO_2_. Thus, HPOX nanoparticles effectively release HBA, which in turn inhibits the production of nitric oxide (NO), thus revealing their ability as a targeted drug delivery system. HPOX has prospects for widespread applicability due to its biodegradable and biocompatible properties (Park et al., 2010; Lee D. et al., 2013). Wu et al. synthesized a PH/ROS dual-responsive triblock polymer (PRDSP) *via* Cu (I)-catalyzed azide–alkyne cycloaddition click polymerization to increase the sensitivity of peroxalate-based nanoparticles. PRDSP is characterized by a disulfide bond, triazole, and peroxalate ester structure in the backbone chain, which allows PRDSP to be cleaved by acid and H_2_O_2_ (Wu et al., 2015). DOX is quickly released from DOX-loaded PRDSP nanoparticles in the presence of GSH (10 mM) and H_2_O_2_ (10 mM), and thus, under acidic conditions, DOX release would be accelerated.

### Proline Oligomer

The side chains of several amino acids in proteins are easily oxidized by ROS to produce carbonyl derivatives, mainly including proline, cysteine, methionine, tyrosine, histidine, and asparagine, and the chains that are the main backbone of proteins are fragmented to form carbonyl derivatives (Garrison, 1987; Stadtman and Levine, 2003; Lee et al., 2014). Proline is one of the most important amino acids that can undergo oxidation for degradation, as it can produce tertiary amide bonds with relatively stronger oxidation properties. Among all ROS-responsive functional groups, the proline oligomer exhibits a relatively slower degradation rate; thus, the proline oligomer was often used for sustained drug release preparations (oxidation-responsive polymeric scaffolds) on the basis of this oxidation-induced cleavage property (Ye et al., 2019). Sung et al. prepared porous polymeric scaffolds *via* terpolymer composed of PEG, poly(ε-caprolactone) (PCL), poly(carboxyl-ε-caprolactone) (cPCL), and proline oligomers as cross-linkers. The properties of polymeric scaffolds could be tuned by the ratios of the PEG, PCL, and cPCL chain segments. The scaffolds selected 4% PEG, 86% PCL, and 10% cPCL could slow degradation for over 20 weeks in conditions containing H_2_O_2_ (Yu et al., 2011).

### Ferrocene-Containing Polymers

Ferrocene is a prototypical metallocene, a type of organometallic chemical compound, consisting of two cyclopentadienyl rings bound on opposite sides of a central metal atom. The rapid interest in organometallic chemistry is attributed to the excitement arising from the discovery of ferrocene and its many analogs due to its reversible redox activity, stability, and ease of synthesis. Hydrophobic ferrocene groups are rapidly quickly oxidized into hydrophilic ferricinium; this phase transition characteristic has been employed to trigger ROS-responsive drug release. For example, Xu et al. synthesized ferrocene-containing amphiphilic block polymers (PACMO-b-PAEFC) by atom transfer radical polymerization (ATRP), with poly(N-acryloylmorpholine) (PACMO) as the hydrophilic blocks and poly(2-acryloyloxyethyl ferrocenecarboxylate) (PAEFC) as the hydrophobic blocks. PACMO-b-PAEFC micelles showed a high PTX encapsulation efficiency of 61.4% and swelled to release the PTX in the presence of ROS. The PTX release rate was mediated by the type and concentration of oxidants. In addition, PACMO-b-PAEFC exhibited low toxicity even at high concentrations of approximately 500 μg ml^−1^ (Xu F. et al., 2017).

## Conclusion and Future Challenges

In the past two decades, many novel ROS-responsive polymers for drug delivery systems have been described, and their structure and function are constantly being improved. ROS-responsive polymers for drug delivery systems have been recognized as a valuable strategy to control drug delivery with low toxicity. Due to the differences in the characteristics of the tumor microenvironment compared to normal cells, ROS-responsive polymer delivery systems meet the theoretical requirements of targeted therapy. With the addition of different reducing structures or functional groups, ROS-responsive polymer systems are moving in the direction of lower toxicity and higher efficiency.

Nonetheless, biocompatible and biodegradable features of different polymers must be improved. If drug delivery carriers are not metabolized or degraded, a large number of carriers will eventually accumulate in normal tissue and inevitably result in damage to normal cell functions, with the possibility of inducing unpredictable systemic toxicity and side effects. Thus, degradable polymers in drug delivery applications had become a prominent research interest due to their biocompatible and degradable properties (Kamaly et al., 2016). The degradable polymers break down inside the body to produce nontoxic natural by-products such as water and carbon dioxide, which are easily eliminated.

Meanwhile, it is urgent to improve the delivery efficiency of polymer carriers. It should be noted that although the concentration of ROS in tumor cells is higher than that in normal cells, ROS levels are still insufficient to fully activate the currently available ROS-sensitive carriers. Thus, delivery systems that respond to multiple stimuli have become a popular strategy of current research because they are safer and achieve superior targeting than delivery systems directed at one stimulus (Roy et al., 2010; Zhang Q. et al., 2017; Cheng et al., 2019; Ma B. X. et al., 2020). However, combining multiple functions into one delivery system remains a challenge. A second excellent approach involves amplification of the intracellular oxidative stress level to improve the response toward polymer nanocarriers. Intracellular oxidative stress could be selectively amplified by the introduction of ROS production agents or *via* the inhibition of intracellular antioxidant systems. (Li J. et al., 2020; Zhang et al., 2020) Furthermore, more functional polymer carriers can be engineered by introducing specific functional building blocks and by applying a design rationale favoring ROS-responsive polymers for drug delivery systems, which not only serve as carriers to deliver active drugs but also exert synergistic effects with the drug and greatly improve the therapeutic outcome of the drug (Ma S. et al., 2020).

Although there are many challenges to overcome (El-Sawy et al., 2018; Rao et al., 2018; Kwon et al., 2019), we expect more innovative ideas and original ideas in this field. Through systematic and in-depth research and careful scientific evaluation, ROS-responsive polymers for drug delivery are expected to provide new opportunities and wider application for the specific treatment of tumors and other diseases.
